# Normal Calcium-Activated Anion Secretion in a Mouse Selectively Lacking TMEM16A in Intestinal Epithelium

**DOI:** 10.3389/fphys.2019.00694

**Published:** 2019-06-13

**Authors:** Génesis Vega, Anita Guequén, Malin E. V. Johansson, Liisa Arike, Beatriz Martínez-Abad, Elisabeth E. L. Nyström, Paolo Scudieri, Nicoletta Pedemonte, Pamela Millar-Büchner, Amber R. Philp, Luis J. Galietta, Gunnar C. Hansson, Carlos A. Flores

**Affiliations:** ^1^Centro de Estudios Científicos (CECs), Valdivia, Chile; ^2^Universidad Austral de Chile, Valdivia, Chile; ^3^Department of Medical Biochemistry, University of Gothenburg, Gothenburg, Sweden; ^4^Telethon Institute of Genetics and Medicine (TIGEM), Pozzuoli, Italy; ^5^Istituto Giannina Gaslini, Genoa, Italy; ^6^Department of Translational Medical Sciences (DISMET), University of Naples Federico II, Naples, Italy

**Keywords:** TMEM16A, cystic fibrosis transmembrane conductance regulator, epithelial transport, colon, intestinal mucus

## Abstract

Calcium-activated anion secretion is expected to ameliorate cystic fibrosis, a genetic disease that carries an anion secretory defect in exocrine tissues. Human patients and animal models of the disease that present a mild intestinal phenotype have been postulated to bear a compensatory calcium-activated anion secretion in the intestine. TMEM16A is calcium-activated anion channel whose presence in the intestinal epithelium is contradictory. We aim to test the functional expression of TMEM16A using animal models with *Cftr* and/or *Tmem16a* intestinal silencing. Expression of TMEM16A was studied in a wild type and intestinal *Tmem16a* knockout mice by mRNA-seq, mass-spectrometry, q-PCR, Western blotting and immunolocalization. Calcium-activated anion secretion was recorded in the ileum and proximal colon of these animals including intestinal *Cftr* knockout and double mutants with dual *Tmem16a* and *Cftr* intestinal ablation. Mucus homeostasis was studied by immune-analysis of Mucin-2 (Muc2) and survival curves were recorded. *Tmem16a* transcript was found in intestine. Nevertheless, protein was barely detected in colon samples. Electrophysiological measurements demonstrated that the intestinal deletion of *Tmem16a* did not change calcium-activated anion secretion induced by carbachol or ATP in ileum and proximal colon. Muc2 architecture was not altered by *Tmem16a* silencing as was observed when *Cftr* was deleted from mouse intestine. *Tmem16a* silencing neither affected animal survival nor modified the lethality observed in the intestinal *Cftr*-null mouse. Our results demonstrate that TMEM16A function in the murine intestine is not related to electrogenic calcium-activated anion transport and does not affect mucus homeostasis and survival of animals.

## Introduction

Electrogenic intestinal anion secretion relies on the concerted action of apical and basolateral membrane proteins. Chloride and bicarbonate accumulate in the intracellular milieu by the activity of basolateral co-transporters and exchangers that provide the required driving force for anion secretion at the apical membrane. Apical exit of anions has two components in many epithelial cells: one component depends on cAMP whereas the other one is controlled by Ca^2+^. While the cAMP-dependent secretion is occurring through CFTR, there is still controversy about the existence and role of a non-CFTR Ca^2+^-activated chloride secretion in the intestine.

Extensive information about Ca^2+^-activated anion secretion has been gained through studies of cystic fibrosis. Cystic fibrosis is the most common human genetic disease; anion secretion is severely decreased due to mutations in the *Cftr* gene affecting mainly airway function, but an important number of patients are also affected by intestinal problems with mucus attachment and obstruction (Gustafsson et al., 2012; De Lisle and Borowitz, 2013). In some patients the existence of Ca^2+^-dependent anion secretion correlates with less severe intestinal symptoms (Bronsveld et al., 2000). Surprisingly, the CF mouse is not affected by muco-obstructive airway disease mostly due to the existence of an active Ca^+2^-activated chloride conductance (Grubb and Boucher, 1999), known to correspond to the TMEM16A protein (Ousingsawat et al., 2009; Rock et al., 2009; Gianotti et al., 2016). Nevertheless, CF-mice are severely affected by intestinal obstructive disease and importantly, the survival of the animals is favored by the existence of intestinal Ca^+2^-activated chloride secretion, that might correspond to a modifier gene of intestinal CF (Rozmahel et al., 1996; Wilschanski et al., 1996; Bleich et al., 2007).

Experiments in the *Tmem16a*^-/-^ mouse pups have shown that there is a reduction in the intestinal Ca^2+^-dependent chloride secretion (Ousingsawat et al., 2009). Similar results were observed when *Tmem16a* was specifically silenced from the intestinal epithelium of adult mice (Schreiber et al., 2015) and, more recently, calcium-activated chloride secretion has been reported to be decreased concomitantly to TMEM16A expression in mouse colon (Rottgen et al., 2018). Nevertheless, we and others have determined that both cAMP and Ca^+2^-activated chloride secretion are occurring via CFTR exclusively in both human and mouse intestine (Seidler et al., 1997; Mall et al., 2000a,b; Zdebik et al., 2004; Bijvelds et al., 2009; Flores et al., 2009; Philp et al., 2018). Intestinal expression of the TMEM16A protein is also a controversial matter, as expression has been reported across the colon epithelium in different portions of the organ (Ousingsawat et al., 2011; Faria et al., 2014; Schreiber et al., 2015). Finally, subcellular expression has been shown to correspond at apical and basolateral membrane locations of intestinal epithelial cells (Yu et al., 2010; He et al., 2011; Ousingsawat et al., 2011; Benedetto et al., 2017; Rottgen et al., 2018).

To test if TMEM16A is expressed and correspond to an important player in Ca^2+^-dependent chloride secretion in the intestine we used the *Tmem16a*^fl/fl^ mouse mated with the *Villin*^cre/-^ mouse to generate an intestinal *Tmem16a* knock-out mouse line and study its function. To evaluate if *Tmem16* corresponds to a modifier gene of intestinal CF disease we crossed the intestinal *Tmem16a* knock-out animal with the *Cftr*^fl/fl^ mouse to generate an intestinal double mutant the *Cftr*^fl/fl^/*Tmem16a*^fl/fl^/*Villin*^cre/-^ mouse. We observed that lethality of the *Cftr*^-/-^/*Villin*^cre/-^ mouse was not altered by the concomitant deletion of *Tmem16a* in the intestinal epithelium. We also evaluated short-circuit currents in ileum and colon samples and found that there were no changes in the magnitude of the currents of the *Tmem16a*^-/-^/*Villin*^cre/-^ intestine when compared to controls. Mucus homeostasis was also unaltered in the *Tmem16a*^-/-^/*Villin*^cre/-^, but severely affected in the *Cftr*^-/-^/*Villin*^cre/-^ intestines independently of the deletion of *Tmem16a* gene.

## Materials and Methods

### Reagents

All chemicals were from Sigma-Aldrich unless stated.

### Animals and Tamoxifen Treatment

The Villin-Cre (*Villin*^cre/-^) mouse was obtained from The Jackson Laboratories. The *Cftr* conditional null mouse (*Cftr*^fl/fl^) (Hodges et al., 2008) and *Tmem16a* mouse (*Tmem16a*^fl/fl^) (Faria et al., 2014) were obtained from their respective laboratories of origin. These animals were housed and mated at CECs animal facility and, male and female animals were used. All experimental procedures were approved by the Centro de Estudios Científicos (CECs) Institutional Animal Care and Use Committee. The Ubc-CreERT2 mouse was obtained from The Jackson Laboratories and the *Tmem16a*^fl/fl^ from its laboratory of origin. The mice colonies were maintained in the IRCCS San Martino–IST Animal Facility (Genoa, Italy). Mice, aged 55–60 days old, were exposed to Cre induction by tamoxifen to knockout the *Tmem16a* gene. Briefly, mice were subjected, once every 24 h for a total of 5 consecutive days, to intraperitoneal injection of 100 μL of a 20 mg/ml tamoxifen solution in corn oil. Twenty-four hours following the final injection, mice were returned to their normal animal room, and closely monitored for additional 55 days. The experimental procedures were performed on male mice. The animal studies were carried out in accordance with the approved guidelines; and were reviewed and approved by the licensing and ethical committee of IRCCS San Martino–IST and by the Italian Ministry of Health. All animals were maintained in the C57Bl6/J strain for at least 20 generations. The RedMUC2^98trTg^ mice (Birchenough et al., 2016) on C57Bl/6N background were housed and bred at the SPF unit of the animal facility at the University of Gothenburg. All animal experimental procedures were in full compliance according to Swedish animal welfare and legislation and approved by the Swedish Laboratory Animal Ethical Committee in Gothenburg, Sweden (number 73-15).

### mRNA Isolation and cDNA Synthesis

Wild type mice were killed through cervical dislocation and intestinal tissues were immediately extracted, epithelium and smooth muscles from colon and ileum samples were isolated by blunt dissection and homogenized in 500 μl of Trizol (Invitrogen^TM^, TRIzol^TM^ Reagent). To fragment high-molecular weight cellular components (DNA) and to minimize their presence in the aqueous phase, the tissue was passed 10 times through a sterile needle. For complete dissociation of nucleoprotein complexes, the homogenate was incubated at RT (15–25°C) for 5 min and centrifuged at 12000 × *g* during 10 min at 4°C. For subsequent phase separation, the supernatant was collected and 100 μl of chloroform added. The sample was vortexed vigorously for 15 s and incubated at RT (15–25°C) for 3 min followed by centrifugation at 12,000 × *g* for 15 min at 4°C. The aqueous phase was transferred to new 1.5 ml RNase-free tube. To precipitate the RNA 250 μl of Isopropanol was added to the samples and it was incubated at –20°C overnight. Precipitated RNA was centrifuged at 12,000 × *g* and purified byb adding 500 μl of 75% ethanol, following with centrifugation at 7,500 × *g* at 5 min at 4°C. The dried pellet was resuspended with 35 μl of nuclease free water, and stored at –80°C. DNA contamination was avoided using DNase treatment. Concentration and integrity of the RNA were determined by spectrophotometry. Total RNA was reverse transcribed into cDNA using the Superscript III RTPCR System (Invitrogen) according to the manufacturer’s recommendations. cDNA synthesis was performed on 2 μg RNA, at 70°C. cDNA integrity was checked using specific primers to cyclophilin (Table 1). 50 ng template cDNA was added to the reaction mixture. Cyclophilin amplification was performed starting with a 5 min template denaturation step at 95°C, followed by 30 cycles of denaturation at 95°C for 30 s and combined primer annealing/extension at 55°C. Relative intensity of brightness of ethidium bromide-stained bands resolved on a 1.5% agarose gel was evaluated.

**Table 1:** Primer sequences and annealing temperatures for the results presented in Figure 1A,B and 2A.

Gene	Accession number	Primer Sequence 5′–3′	Annealing Temp. (°C)
*Cftr*	NM_021050.2	Forward: GCCATTTACCTTGGCATAGGC Reverse: GCCAAGGCAAGTCCTTCATCA	63
*Tmem16a*	NM_178642.5	Forward: AGGAATATGAGGGCAACCTG Reverse: CGACACCATGGATTTTGGTA	63
*Cyclophilin (Ppia)*	NM_008907.2	Forward: GGCAAATGCTGGACCAAACACAA Reverse: GTAAAATGCCCGCAAGTCAAAAG	63
*Tmem16a* (Exons 6–16)	NM_178642.5	Forward: AAGAGAACAACGTGCACCAA Reverse: GAAATAGGCTGGGAATCGGT	61.3

### Real Time PCR

Quantification of *Tmem16a, Cftr*, and *Cyclophilin* mRNA expression was performed using SYBR Green detection in a LightCycler PCR machine according to the manufacturer’s instructions.

We determined the PCR efficiency of each individual assay by measuring serial of 100 ng cDNA from a pool of ileum or colon from epithelia or smooth muscle in triplicate. Only CT values < 40 were used for calculation of the PCR efficiency. All PCRs displayed an efficiency between 96 and 100%. Amplifications were performed starting with a 3 min template denaturation step at 94°C, followed by 45 cycles of denaturation at 94°C for 20 s and combined primer annealing/extension at the gene specific primer temperature for 30 s (Table 1). All samples were amplified in triplicate and the mean was obtained for further calculations. Relative-fold changes in target gene expression were quantified by the previously reported ΔΔC_T_ method (Livak and Schmittgen, 2001). Briefly, epithelial and smooth muscle samples were amplified in the same run. C_T_ values were obtained for individual samples using the Rotor-Gene 6000 software 1.7 (Corbett Life Science Pty Ltd., Sydney, Australia), where the targets (*Tmem16a* or *Cftr*) and reference (*Cyclophilin*) had the same cDNA concentration. ΔC_T_ was calculated by subtracting the C_T_ (target – reference).

### Preparation of Epithelial Cells

Freshly collected distal colonic or distal small intestinal tissue from wild type and RedMUC2^98trTg^ mice was flushed with ice-cold HBSSwo (Hank’s balanced salt solution without Mg^2+^/Ca^2+^ supplemented with 10 mM HEPES, pH 7.2) to remove luminal content. The colonic tissue was inverted and inflated by injection of HBSSwo. Epithelial isolation was performed twice by incubation of tissue for 30 min in 20 ml pre-digestion buffer (HBSSwo, 5 mM EDTA, 5% v/v FCS, 1 mM DTT) at 37°C and 140 RPM. The epithelium was detached by vortexing for 30 s and the remaining tissue was discarded. The isolated epithelial cells were centrifuged at 400 RCF for 10 min at 4°C, resuspended in 2 ml digestion buffer (HBSS with Mg^2+^/Ca^2+^ supplemented with 2 mg/ml collagenase type I and 40 U/ml DNase I) and incubated at 37°C for 30 min, with mixing every 10 min. Cells were washed and re-suspended in 1 ml ice-cold PBS. Cells were stained with Fixable Viability Dye eFluor^TM^ 780 (eBioscience^TM^, 1:1000) for 30 min, on ice and washed in 25 ml ice-cold HBSSwo before re-suspension in 1 ml FACS buffer (HBSSwo, 2% v/v FCS, 5 mM EDTA) prior to FACS. Goblet cells and remaining epithelial cells were sorted using a FACS Jazz (Becton Dickinson) according to the presence or absence of mCherry signal. Dead cells were excluded from the sorting using the Fixable Viability Dye eFluor^TM^ 780. In the living population, discrimination of doublets or aggregated cells was consecutively made for SSC (SSC-Width (W)/ SSC-Height (H) plot) and for FSC (FSC-W/FSC-H plot). The purity of the sorted cells was >96% (See Supplemental Figure 1).

### RNA Extraction and Sequencing

Sorted cells were pelleted at 400 RCF for 5 min and immediately resuspended in 350 μl RLT buffer (Qiagen) supplemented with β-merchaptoethanol (Gibco). Samples were homogenized using QIAshredder columns (Qiagen) according to manufacturer’s instructions. Goblet cell lysates were loaded onto RNeasy MinElute column (Qiagen) and remaining epithelial cell lysates onto RNeasy Mini column (Qiagen). The columns were washed according to manufacturer’s instructions and RNA was eluted with 35 μl (remaining epithelium) or 18 μl (goblet cells) RNAse free H_2_O (Qiagen). The quality of isolated RNA was determined using an Experion Automated Electrophoresis platform (Bio-Rad) and samples were kept at –80°C until further analysis.

RNA sequencing was performed by the Genomics Core Facility at the Sahlgrenska Academy (Gothenburg, Sweden). cDNA libraries were prepared with TruSeq Stranded Total RNA Sample Preparation kit with Ribo Zero Gold (Rev. E; Illumina) according to manufacturer’s protocol and sequenced via paired-end sequencing with the NextSeq500 platform (Illumina). The quality for the sequencing was measured for all lanes, reads and cycles with 93.4% of bases above Q30. Quality of the raw data was assessed with the FastQC software (version 0.11.2) looking at per base sequence quality and adapter contamination. The reads were mapped using mouse reference genome mm10 using aligner STAR (version 2.5.2b) and the number of the mapped reads on each gene was calculated by the HTseq software (version 0.6.1p1). Normalization of the data and differential expression were performed using the R package DESeq2 (version 1.14).

### Peptide Preparation and LC-MS/MS

FACS sorted cells were solubilized in 30 μL lysis buffer (4% SDS, 100 mM Tris–HCl pH8, 100 mM DTT), heated 5 min at 95°C and digested with LysC and trypsin on 30 kDa filters (Vivacon 500, Sartorious Biotech) according to FASP protocol (Wiśniewski et al., 2009b). Samples were pooled and fractioned according to the pipette tip SAX protocol (Wiśniewski et al., 2009a) at pH 11, 8, 6, 5, 4 and 3. The remaining peptides were eluted in 50% ACN, 0.5% FA, 0.25 M NaCl. All samples were cleaned up with C18 StageTips (Rappsilber et al., 2007).

Peptides were analyzed with an EASY-nLC 1000 system (Thermo Fisher Scientific) connected to a Q-Exactive mass-spectrometer (Thermo Fisher Scientific) through a nanoelectrospray ion source. SAX fractionated peptides were analyzed with a reverse-phase column (150 mm × 0.075 mm inner diameter, New Objective, Woburn, MA) packed in-house with Reprosil-Pur C18-AQ 3 μm particles (Dr. Maisch, Ammerbuch, Germany), using 120 min gradient, 0–25% B for SAX pH 11 fraction, 5–40% B for SAX fractions pH 8, 6, 5, 4, and 3, 5–60% B for final elution of SAX columns (A: 0.1% formic acid, B: 0.1% formic acid/80% acetonitrile).

Q-Exactive was operated at 250°C capillary temperature and 2.0 kV spray voltage. Full mass spectra were acquired in the Orbitrap mass analyzer over a mass range from m/z 400 to 1600 with resolution of 70 000 (m/z 200). Twelve most intense peaks with a charge state ≥2 were fragmented in the HCD collision cell with normalized collision energy of 30%, and tandem mass spectrum was acquired in the Orbitrap mass analyzer with resolution of 17 500. Dynamic exclusion was set to 10 s. The maximum allowed ion accumulation times were 60 ms for full MS scans and 64 ms for tandem mass spectrum and AGC targets 5e5 for both, full MS and tandem MS.

MS raw files were processed with MaxQuant software version 1.5.7.4 (Cox and Mann, 2008) by searching against mouse UniProt database (canonical database, downloaded 20180611, containing 53 444 proteins). Searches were performed with full tryptic specificity, maximum 2 missed cleavages, carbamidomethylation of cysteine was set as a variable modification, methionine oxidation and protein N-terminal acetylation were set as variable modifications. False discovery rate (FDR) was set to 1% both, for peptide and protein levels and the minimum required peptide length was set to 6 amino acids. Quantification was done based on LFQ protocol included in the MaxQuant search.

### Immunoblotting

Smooth muscle and epithelial samples of ileum and proximal colon obtained from wild type and *Tmem16*^fl/fl^/*Villin*^cre/-^ animals were obtained by blunt dissection and resuspended in 200 μl of RIPA buffer 1× (50 mM Tris–HCl pH 7.4, 150 mM NaCl, 1% Nonidet P-40, 0.5% sodium deoxycholate, 0.1% SDS, 1 mM Na3VO4, 1 mM PMSF) containing Complete Protease Inhibitor Cocktail. Tissue was homogenized using sonication and then centrifuged at 16,000 × *g* for 30 min at 4°C. Protein concentration for each individual sample was determined using the Pierce BCA protein quantification kit (Thermo Fisher Scientific, Waltham, MA). Samples were electrophoresed (30 μg per lane) with Tris–Glycine 4–20% gel and transferred to a nitrocellulose membrane (Bio-Rad, Hercules, CA, United States) for Western blotting. Nitrocellulose membranes were cut in two pieces using the 75 kDa standard ladder as reference, and both pieces were blocked with 5% BSA in Tris-buffered saline including 0.1% Tween 20 (TBST) for 1 h at room temperature. The upper membranes were incubated overnight with TMEM16A primary antibody 1:1000 dilution (Abcam Cat# ab64085, RRID:AB_1143505) or a rabbit polyclonal antibody for TMEM16A raised against the N-terminus of mouse protein (RVPEKYSTLPAEDR) as described previously (Huang et al., 2009). After washing three times with TBST, the blots were further incubated for 60 min at room temperature with an anti-rabbit secondary antibody (1:20000 dilution Santa Cruz Biotechnology, CA). In parallel the lower membranes were incubated for 40 min with β-actin antibody (C-4) HRP (1:8000 dilution, Santa Cruz Biotechnology Cat# sc-47778 HRP, RRID:AB_2714189). Both membranes were washed three times with TBST and then visualized using the ECL (SuperSignal West Femto Maximum Sensitivity Substrate, Thermo Fisher Scientific).

### Immunolocalization of TMEM16A and MUC-2

Histological sections of mouse intestine were deparaffinized and subjected to antigen retrieval with 0.5% SDS in PBS for 15 min. After permeabilization with 0.3% Triton X-100 in PBS for 5 min, samples were blocked with 1% bovine serum albumin (BSA) in PBS for 2 h and then incubated overnight at 4°C with an anti-TMEM16A antibody (Santa Cruz Biotechnology Cat# sc-69343, RRID:AB_2058312) diluted 1:50 in PBS containing BSA 1%.

Following incubation with primary antibody, tissues were rinsed three times in PBS and incubated with goat anti-rabbit Alexa Fluor 488 secondary antibody (Life Technologies) diluted at 1:200 in 1% PBS-BSA for 1 h protected from the light. After further 3 washes in PBS, slides were mounted with Fluoroshield with 4′,6-diamidino-2-phenylindole (DAPI) (Sigma-Aldrich) to stain cell nuclei. Confocal microscopy was performed using a laser scanning confocal microscope TCS SPE (Leica Microsystems). Image analysis was performed using Leica and ImageJ software. Immunlocalization of TMEM16A in human tissues was performed using the SP31 antibody 1:200 dilution. To detect bound antibodies, we used the LSAB Universal Kit (Dako). The positive reaction was developed with diaminobenzidine (Vector Labs) and sections were counterstained with hematoxylin. The protocols to isolate, culture, store, and study tissues from patients were approved by the Regional Ethical Committee (Comitato Etico Regionale) under the supervision of the Italian Ministry of Health (registration number: ANTECER, 042-09/07/2018). Informed and written informed consent was obtained using a form that was also approved by the same Ethical Committee.

Intestinal sections were fixed in freshly prepared, ice-cold Methacarn (methanol-Carnoy) solution (60% absolute methanol, 30% chloroform, 10% glacial acetic acid), Tissues were washed in dry methanol (2 × 30 min), absolute ethanol (2 × 15 min), absolute ethanol/ xylene 1:1 (1 × 15 min) and in xylene (2 × 15 min), then waxed, paraffin embedded with vertical orientation and cut in 4 μm thick sections. Paraffin embedded sections were dewaxed with Xylene substitute and rehydrated before antigen retrieval with 10 mM citric acid, pH6. Immunostaining was performed with rabbit anti-MUC2-C3 (1:1000) (Johansson et al., 2008) or anti-Muc2 apomucin (PH 497) (1:1000) (Hansson et al., 1994). The staining was detected with goat anti-rabbit IgG conjugated to Alexa Fluor^®^ 488 (Molecular Probes^®^, Thermo Fisher Scientific, 1:2000). DNA counter staining was carried out for 5 min using 1 μg/ml Hoechst 34580 (Molecular Probes^®^, Thermo Fisher Scientific). Pictures were obtained by using a Nikon eclipse E1000 fluorescence microscope (Nikon) with 40×/0.75 or 20×/0.5 Plan Fluor objectives. Images (2–3 sections analyzed per animal) were acquired using the NIS elements software (Nikon). Samples were shipped from the Chilean lab to the Swedish lab and genotypes disclosed after staining and analysis.

### Ussing Chamber Measurements

Stripped colon samples and intact ileum samples were placed in 0.1 cm^2^ surface area tissue-holders in modified Ussing chambers (Physiologic Instruments, United States). Tissues were bathed in bicarbonate-buffered solution (pH 7.4) of the following composition (in mM): 120 NaCl, 25 NaCO_3_, 3.3 KH_2_PO_4_, 0.8 K_2_HPO_4_, 1.2 MgCl_2_, 1.2 CaCl_2_ and 10 D-glucose gassed with 5%CO_2_+95%O_2_ and kept through the experiment at 37°C. The transepithelial potential difference referenced to the basolateral side was measured using a VCC MC2 amplifier (Physiologic Instruments, United States) under current clamp mode. Short 200 ms 10 μA pulses were given every 1 s. The short-circuit currents were calculated using the Ohm’s law as previously described. After 10–15 min of incubation the tissues were stimulated with either 100 μM carbachol (CCh) or 100 μM ATP added on the basolateral side to induce calcium-activated anionic secretion. The magnitude (ΔI_sc_) of the currents was calculated as the difference after and before stimulation as described (Flores et al., 2010).

### Statistical Analysis

Unless otherwise stated all values corresponds to mean ± SEM. All data were analyzed using Sigmaplot 12.3 software. The tests used are described on each figure legend or in the main text when corresponds.

## Results

### *Tmem16a* Transcripts but Not Protein Is Detectable in Mouse Intestinal Epithelium

**FIGURE 1:**
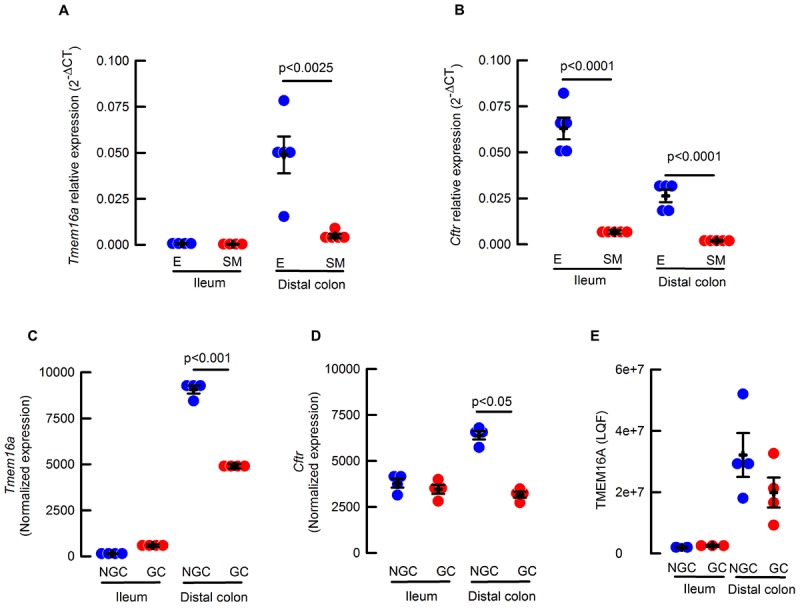
**(A)** *Cftr* and **(B)** *Tmem16a* transcripts isolated from epithelium (E, blue circles) or muscle (SM, red circles) of ileum and proximal colon; Values correspond to 2^-ΔΔCT^ *Tmem16a* **(A)** or *Cftr* **(B)**, normalized to *Cyclophilin* and relative to epithelium; *n* = 4–5 for each group and *p* indicates the corresponding *t*-test value. **(C)** *Tmem16a* expression from RNAseq analysis of sorted goblet (GC, red circles) and non-goblet cells (NGC, blue circles) in ileum and distal colon; *n* = 4 per group. **(D)** *Cftr* expression from RNAseq analysis of sorted goblet (GC, red circles) and non-goblet cells (NGC, blue circles) in ileum and distal colon; *n* = 4 per group. **(E)** Abundance of TMEM16A protein by proteomics using label free quantification of sorted goblet (CG, red circles) and non-goblet cells (NGC, blue circles) in ileum and distal colon *n* = 2–4 per group.

To analyze the expression of TMEM16A on intestinal tissues we started determining mRNA levels in epithelial and smooth muscle tissue samples. We reasoned that the smooth muscle was a good control since it is known to express functional TMEM16A channels (Huang et al., 2009), this tissue section also includes Cajal cells with demonstrated high functional expression of TMEM16A (Gomez-Pinilla et al., 2009; Sanders et al., 2012). Interestingly, only proximal colon epithelium showed significantly larger mRNA expression than its smooth muscle counterpart (Figure 1A). When epithelial samples were compared, proximal colon expressed a larger amount of *Tmem16a* transcripts compared to ileum. Levels of *Cftr* mRNA were also determined and showed to be significantly higher in both ileum and colon epithelium when compared to the corresponding smooth muscle layer (Figure 1B).

It has been shown that goblet cells express calcium-activated anion channels in colon (Yu et al., 2010) and airway (Scudieri et al., 2012) goblet cells. So, a more detailed analysis of *Tmem16a* and *Cftr* mRNA expression was performed in goblet and enterocytes (non-goblet) isolated from intestinal epithelium. As observed in Figure 1C, *Tmem16a* was almost absent in ileum but significantly higher in distal colon (*p* < 0.001 one way ANOVA), and in the case of colon, more abundant on enterocytes than goblet cells (Figure 1C). Expression of *Cftr* mRNA was detected in both ileum and distal colon epithelial and goblet cells with similar variation between the groups as seen for *Tmem16a* expression (Figure 1D). Finally, analysis of the proteome detected very low levels of TMEM16A in ileum, but showed significantly higher expression of TMEM16A in distal colon, a level that still could be considered a low when compared to other proteins like the triple-cotransporter NKCC1 (4.2e+8 ± 8e+7 and 3.5e+8 ± 3e+7 LQF; *n* = 4 for both data sets) in goblet cells from ileum and colon, respectively. In summary, we found a higher expression of mRNA and protein for TMEM16A in colon than ileum in mouse.

**FIGURE 2:**
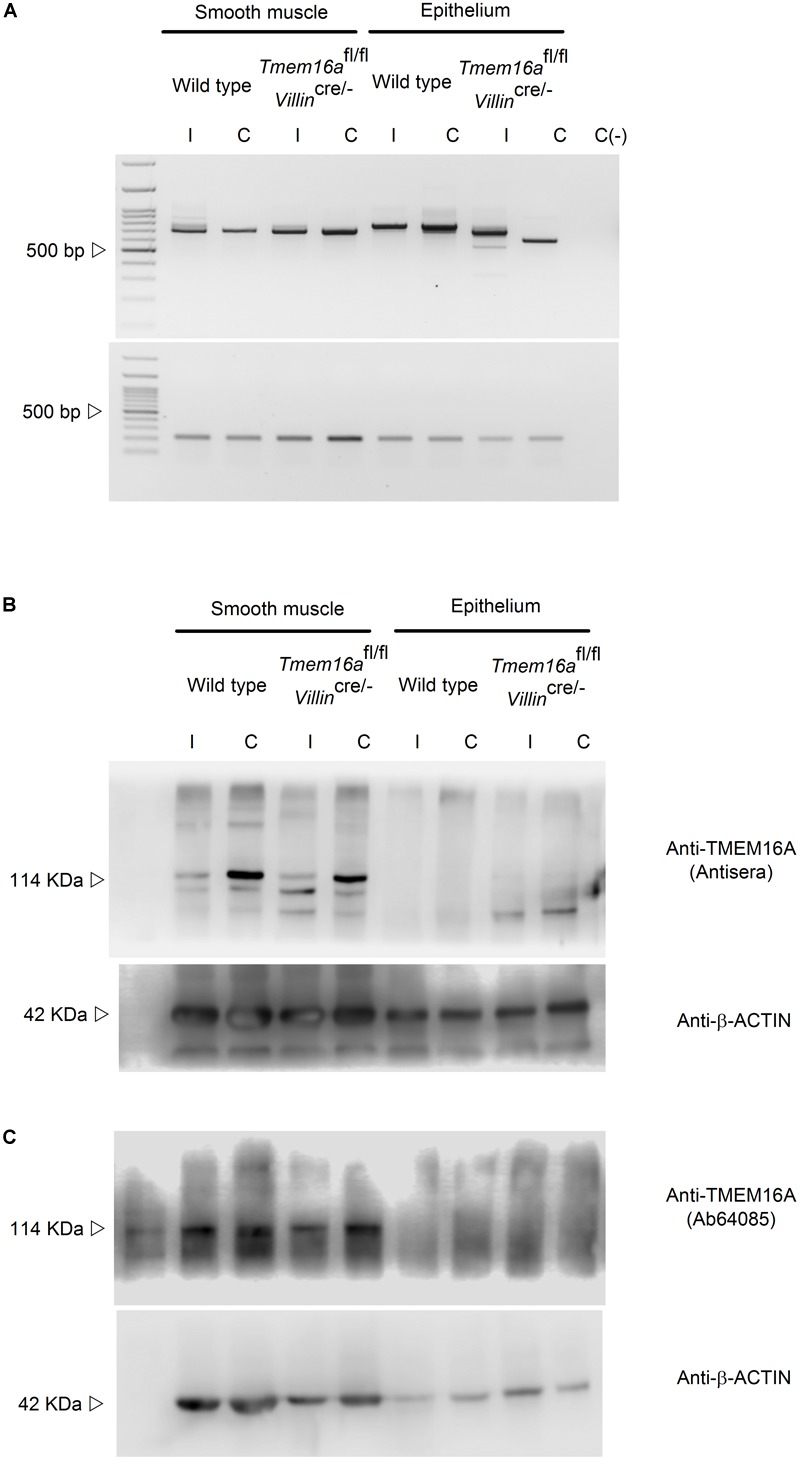
TMEM16A is not expressed in intestinal epithelium in mouse. **(A)** cDNA screening for exon 12 deletion after recombinase activity in the *Tmem16a*^fl/fl^/*Villin*^cre/-^ mouse. Representative image of 3 analyzed animals per group. **(B,C)** TMEM16A protein expression in intestine was tested in smooth muscle and epithelium of ileum (I) and proximal colon (C) of wild type and *Tmem16a*^fl/fl^/*Villin*^cre/-^ animals. Expected molecular weight for TMEM16 is 114 kDa and 42 for β-actin. Representative image of 3 experiments.

Next we used the intestinal-specific *Tmem16*-null animals (*Tmem16*^fl/fl^/*Villin*^cre/-^) to test TMEM16A expression. To confirm the specific deletion of exon 12 from the *Tmem16a* gene, mRNA isolated from epithelium and smooth muscle was retro-transcribed and screened by PCR with primers harboring exons 6 to 16 of the gene. Wild type Ileum presented a PCR product of ∼800 bp. After deletion of exon 12 (83 bp) a band between 700 and 750 bp was noticeable. Mouse proximal colon was rich in a ∼750–800 bp PCR product that was not present in the *Tmem16*^fl/fl^/*Villin*^cre/-^ animals and a ∼650 bp band appears instead. A ∼700 bp PCR product was amplified from muscle in ileum and proximal colon and found to remain unaltered in the *Tmem16*^fl/fl^/*Villin*^cre/-^ mouse (Figure 2A).

Finally, immunoblot detection of TMEM16A protein was performed with 2 different antibodies. On both occasions we observed nearly non-detectable protein in epithelial samples from ileum and proximal colon, while smooth muscle showed a constant detectable amount of TMEM16A protein in all samples (Figure 2B,C). The double band observed with both antibodies has also been detected by others when using different antibodies (Yu et al., 2010; Mroz and Keely, 2012; Rottgen et al., 2018). This might account for the diversity of TMEM16A variants expressed in intestinal tissues (O’Driscoll et al., 2011).

To further explore intestinal TMEM16A expression we attempted immunofluorescence detection on intestinal tissues. Positive staining was observed in the cytoplasm and few cells stained with apical membrane in both ileum and proximal colon of the wild type animals. Nevertheless, similar staining was observed in the *Tmem16a*^fl/fl^/*Villin*^cre/-^ samples (Supplemental Figures 2A–D). These results indicate that the antibody used for the staining cannot correctly recognize the murine protein in the intestine. Finally, we immuno-localized TMEM16A in human tissue samples, and positive reaction was found in cells that resemble those of interstitial Cajal cells but no staining was observed on the epithelial layer (Supplemental Figures 2E,F).

### TMEM16A Does Not Participate in Anion Secretion of the Mouse Intestine

**FIGURE 3:**
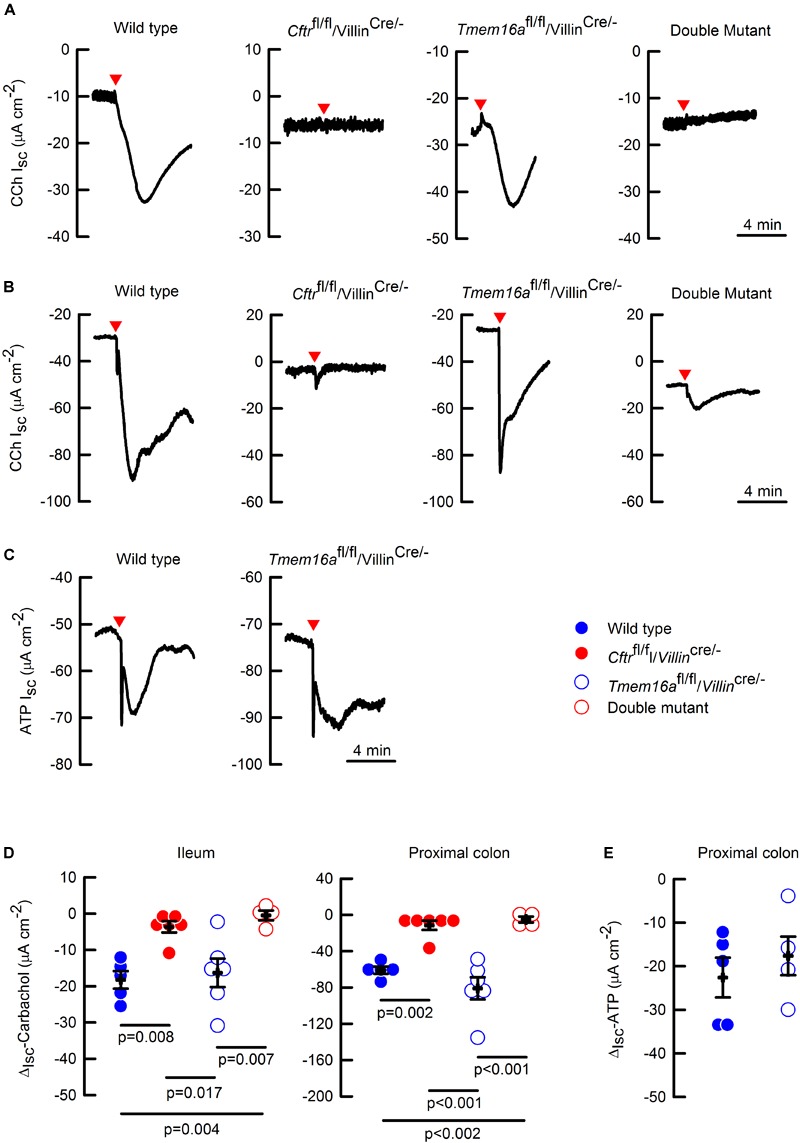
*Cftr* but not *Tmem16a* silencing abolished calcium-activated anion secretion in mouse intestine. Representative images of CCh-induced short-circuit currents (Isc) from in the ileum **(A)** and proximal colon **(B)** of the different mice. **(C)** Representative images of ATP-induced short-circuit currents in the wild type and *Tmem16a*^fl/fl^/*Villin*^cre/-^ mice. Red arrowheads indicate the time of CCh (100 μM) or ATP (100 μM) addition on the basolateral side for each image. Short-circuit current changes (ΔI_sc_) in ileum and colon induced by CCh **(D)**, and in colon induced by ATP **(E)**, are summarized; *n* = 4–6 for CCh in ileum and colon on each group; *n* = 5 for wild type and *n* = 4 for *Tmem16a*^fl/fl^/*Villin*^cre/-^ in the ATP experiments. Indicated *p* values correspond to one way ANOVA **(D)** or unpaired *t*-test **(E)** analysis. No significant difference found for groups in panel **(E)**.

To test if TMEM16A participates in the electrogenic anion secretion of the intestine, we performed Ussing chamber experiments on ileum and proximal colon samples of mice including *Cftr*^fl/fl^/*Villin*^cre/-^, and *Cftr*^fl/fl^/*Tmem16a*^fl/fl^/*Villin*^cre/-^ double mutants. Even though, we did not find any differences in carbachol (CCh) activated anion secretion between proximal and distal colon (50.9 ± 4 vs. 58.3 ± 9 μA cm^-2^, respectively; *n* = 3 per group; *p* > 0.05 *t*-test), we chose to work with proximal colon as it has been previously shown to present stronger responses to calcium agonists (Schreiber et al., 2015). As shown in Figure 3, CCh stimulation induced a rapid change in I_sc_ interpreted as anionic secretion in ileum (Figure 3A) and proximal colon (Figure 3B) of wild type animals. Silencing of *Tmem16a* did not affect CCh-response in any of the tissues. Nevertheless, intestinal silencing of the *Cftr* gene almost ablated the CCh response of the intestinal epithelium. Interestingly, the double mutant tissues showed similar responses as the *Cftr*^fl/fl^/*Villin*^cre/-^ intestine discarding a further effect of TMEM16A on intestinal anion secretion.

To discard the possibility that carbachol signaling is uncoupled from TMEM16A activation in the intestine, we tested the effect of a different calcium agonist. We chose ATP that is known to activate TMEM16A (Namkung et al., 2011b), and observed that ATP-induced anion secretion was not affected by *Tmem16a* silencing in proximal colon (Figure 3C). The CCh and ATP experiments are summarized in Figure 3D,E, respectively.

### Lack of CFTR but Not TMEM16A Results in Mucus Disruption and Bacterial Accumulation in Mouse Intestine

**FIGURE 4:**
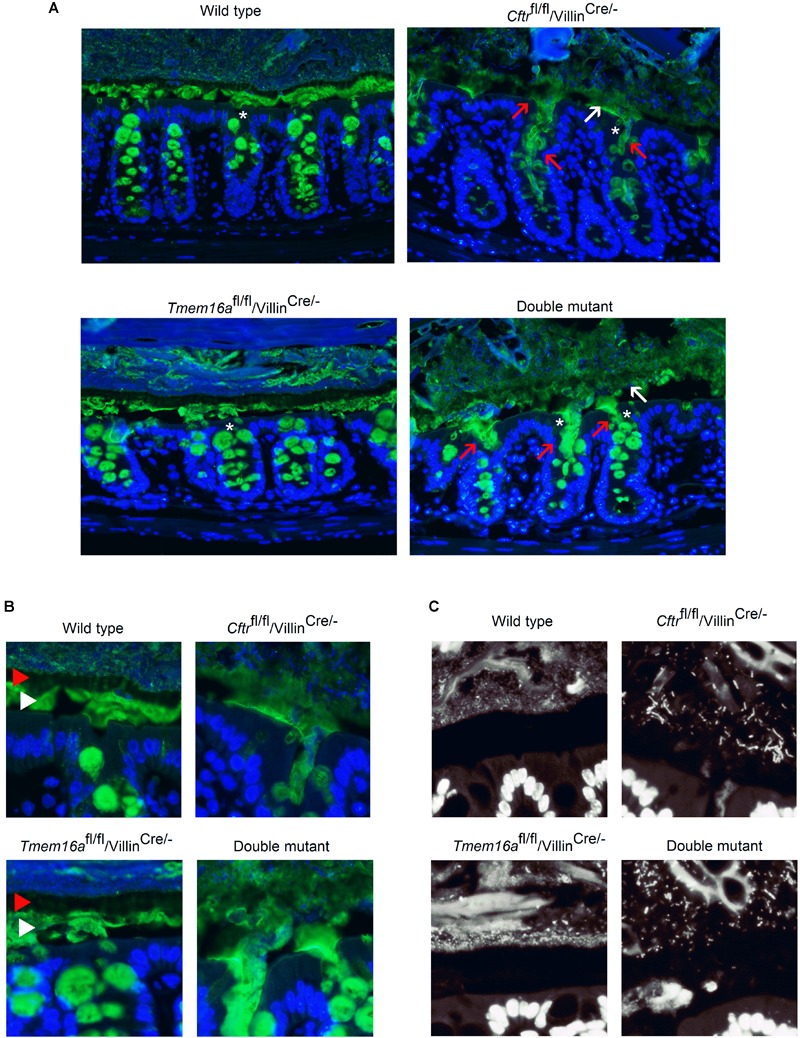
Mucus homeostasis and bacterial accumulation is not affected by *Tmem16a* silencing in the proximal colon of mouse. **(A)** Representative images of Muc2 staining (green) in Carnoy’s fixed colon samples. Red arrows indicate massive release of mucus from the crypts and white arrows indicate poorly structured mucus layer. Blue staining corresponds to Hoescht nuclear DNA. White asterisks indicate crypts that are amplified in panel **(B)**. **(B)** Images show detailed mucus structured in clearly defined external layer (red arrow head) and internal layer (white arrow head). Layers remain conserved in the *Tmem16a*^fl/fl^/*Villin*^cre/-^ tissues, but organization is lost when *Cftr* is silenced. **(C)** *Cftr* silencing shows the presence of bacteria accumulation independently of *Tmem16a* expression. Some bacteria can be seen invading the mucus layers; *n* = 5 animals for each group and 2–3 fields were analyzed for each animal.

Mucus homeostasis is tightly related to anion secretion in the intestine (Gustafsson et al., 2012). To test if TMEM16A deficiency affects mucus homeostasis we performed Muc2 mucin staining on fixed intestinal tissues. As observed in Figure 4A mature Muc2 was massively released from the crypts and the mucus layer was unstructured in *Cftr*^fl/fl^/*Villin*^cre/-^. The same pattern was observed in samples obtained from the *Cftr*^ΔF508/ΔF508^ mouse (Supplemental Figure 3A), confirming that *Cftr* silencing in intestinal epithelium is sufficient to cause the mucus alterations. The insets shown in Figure 4B show that mucus organizes in outer and inner layers, but such organization is lost when *Cftr* is silenced. We next analyzed colon samples from the *Tmem16a*^fl/fl^/*Villin*^cre/-^ animals and found no changes in mucin appearance compared to wild types, but the analysis of the double mutant tissues showed similar mucus alterations as observed in *Cftr*^fl/fl^/*Villin*^cre/-^ mouse (Figure 4A). Control staining in the colon of *Villin*^cre/-^ animals showed no alterations in their mucus structure (Supplemental Figure 3A).

A similar pattern of alterations was observed in ileum samples stained for mature Muc2. After *Cftr* silencing Muc2 accumulates in the crypts and between villi (Supplemental Figure 3B). This was not the case when *Tmem16a* was silenced, as there are no detectable differences in the staining of mature Muc2 when compared to wild type tissues. Samples obtained from the double mutant animals showed the same features as the *Cftr*^fl/fl^/*Villin*^cre/-^ animals. Additional analysis of immature Muc2 (Muc2-apomucin) staining, normally located in the intracellular compartment, showed no extracellular accumulation after *Cftr* and/or *Tmem16a* silencing in colon and ileum (Supplemental Figure 4).

Another common phenomenon that affects CF mice and patients is the overgrowth of bacteria in the intestine (Norkina et al., 2004; Dorsey and Gonska, 2017). Using DNA staining we observed that samples from the colon of *Cftr*^fl/fl^/*Villin*^cre/-^ and double mutant animals showed increased amount of bacteria in the inner mucus layer, close to the apical membrane of the epithelium when compared to the wild type samples (Figure 4C). In most samples we did not observe bacteria in direct contact with the epithelium or in the mucus of the colon crypts. The samples from *Tmem16a*^fl/fl^/*Villin*^cre/-^ animals had a stratified mucus separating bacteria from the epithelium as seen in wild type mice.

### *Tmem16a* Intestinal Deletion Did Not Affect Survival in the *Cftr*^fl/fl^/*Villin*^cre/-^ Mouse

**FIGURE 5:**
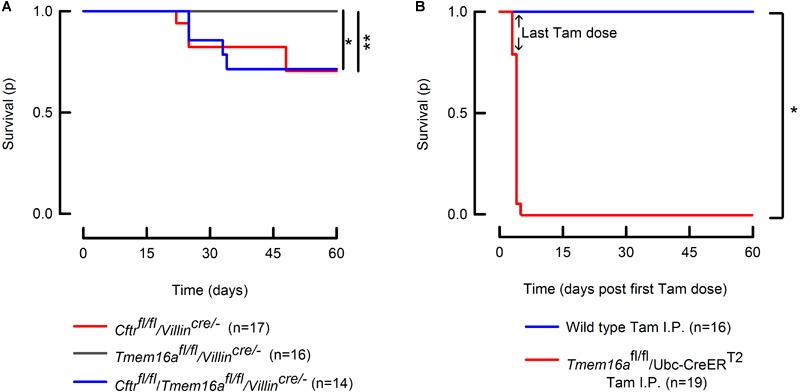
Intestinal silencing of *Tmem16a* is not lethal in the mice. **(A)** Kaplan–Meier curves showing survival of the *Tmem16a*^fl/fl^/*Villin*^cre/-^, *Cftr*^fl/fl^/*Villin*^cre/-^ and *Tmem16a*^fl/fl^/*Villin*^cre/-^ double mutant mice. Intestinal silencing of *Tmem16a* does not alter survival of the *Cftr*^fl/fl^/*Villin*^cre/-^ mouse. Log-Rank test: ^∗^indicates *p* = 0.025 and ^∗∗^*p* = 0.028. **(B)** *Tmem16a* tamoxifen (Tam) inducible silencing produced a lethal phenotype in mice. Arrows indicate the last dose of tamoxifen in both groups. Log-Rank test: ^∗^ indicates *p* < 0.001.

We observed that *Tmem16a* deletion from intestinal epithelium did not alter survival determined up to 60 days of age. In order to evaluate if *Tmem16a* could act as a modifier and might enhance lethality in a model that is already affected by intestinal obstructive disease like the *Cftr*^fl/fl^/*Villin*^cre/-^ mouse. To test this possibility, we recorded survival for the double mutant mice. Our results demonstrate that there are no changes in lethality between the double mutant and *Cftr*^fl/fl^/*Villin*^cre/-^ animals (Figure 5A), discarding a role for TMEM16A in causing lethal intestinal obstruction. Dead mice carcasses that could be recovered for post-mortem examination showed rupture of the small intestine. We studied also the lethality of inducible *Tmem16a*-null mice and found that the phenotype is 100% lethal in adult mice, demonstrating that lethality after *Tmem16a* silencing is due to a severe non-intestinal phenotype.

Finally, we recorded weight of animals at 58–60 days and found that intestinal silencing of *Cftr* produced a body weight reduction independently of *Tmem16a* silencing (Table 2).

**Table 2:** Mice weight is affected after *Cftr* but not *Tmem16a* silencing in mouse intestine.

Genotype	Mean ± S.E.M. (gr)	*n*	vs. Wild type	vs. *Cftr*^fl/fl^/ *Villin*^cre/-^	vs. *Tmem16a*^fl/fl^/ *Villin*^cre/-^
Wild type	22.6 ± 0.7	7	–	*p* = 0.04^∗^	*p* = 0.324
*Cftr*^fl/fl^/*Villin*^cre/-^	19.0 ± 2.1	6	*p* = 0.04^∗^	–	*p* = 0.003^∗^
*Tmem16a*^fl/fl^/*Villin*^cre/-^	24.7 ± 1.5	6	*p* = 0.324	*p* = 0.003^∗^	–
Double mutant	13.5 ± 1.0	6	*p* < 0.001^∗^	*p* = 0.008^∗^	*p* < 0.001^∗^

*Analysis of weight in 58–60 days old mice; *p* values correspond to results after Two-way ANOVA.*

## Discussion

Calcium-activated anion secretion has been postulated as an alternative to CFTR, with important therapeutic implications in cystic fibrosis. The advent of the use of animal models to study CF was marked by the observation that mouse models were severely affected by intestinal and not lung disease (Guilbault et al., 2007). Shortly after generation of the first CF mice (generated by *Cftr* gene knockout or insertion of CF-causing mutations), studies demonstrated that the surviving animals displayed calcium-activated anion currents in diverse tissues including the intestine. This anion conductance was hypothesized to be responsible for a mild CF phenotype and enhanced survival (Clarke et al., 1994; Rozmahel et al., 1996; Wilschanski et al., 1996). Similar observations were also reported in humans, unleashing the hunt for calcium-activated anion channels parallel to CFTR (Veeze et al., 1994). As several genes were proposed to encode the elusive calcium-activated chloride channel it was not until 2008 that TMEM16A was identified as the protein responsible for calcium-activated chloride secretion (Caputo et al., 2008; Schroeder et al., 2008; Yang et al., 2008).

The first attempt to test the role of TMEM16A in the intestinal secretory function was performed in the systemic *Tmem16a*^-/-^ mice. Due to the severe lethal phenotype, causing death of animals in the first days of life, the experiments were performed in distal colon of pups. In this way it was possible to observe an almost completely absence of carbachol-induced chloride secretion, but no reduction in cAMP-induced chloride secretion indicating that the two ion conductive pathways were occurring through different channels (Ousingsawat et al., 2009). Intriguingly, we observed that the *Tmem16a* gene is transcribed in colon epithelium but we were unable to find convincing TMEM16A protein expression using three different antibodies in the mouse (Figure 1A–D) and a fourth in human intestine (Supplemental Figures 2E–F). Several other reports are in accordance with our observations. In one of the earliest efforts to identify the TMEM16A channel in mouse tissues the protein was not detected in intestinal epithelial cells (Huang et al., 2009), and during the identification of interstitial cells of Cajal in human and mouse intestine, TMEM16A was rarely seen in cells of the mucosa, and when found the morphology and location of positive cells corresponded to myofibroblasts and not to epithelial cells (Gomez-Pinilla et al., 2009). In large scale screenings of human tumors, TMEM16A expression was consistently present in gastrointestinal cancers and also in several normal tissues including epithelium of breast, prostate, salivary glands, stomach, pancreas and gallbladder, but not in small or large intestine epithelium (West et al., 2004; Miettinen et al., 2009). TMEM16A expression in human gastrointestinal tumors might also explain the reported activity of TMEM16A on immortalized human intestinal cells (Namkung et al., 2011a; Mroz and Keely, 2012). Moreover, upregulation of the channel expression has been related with increased metastasis in human colorectal tumor cells (Sui et al., 2014), suggesting that TMEM16A expression on the intestine is part of the pathophysiology of intestinal tumorigenesis. Nevertheless, is necessary to point out that other studies show epithelial cell with TMEM16A staining in some cases using the same antibodies tested by ourselves (Yu et al., 2010; He et al., 2011; Ousingsawat et al., 2011; Benedetto et al., 2017; Rottgen et al., 2018). In addition we analyzed the protein content of goblet and non-goblet cells prepared from the intestinal epithelium and we do not find any TMEM16A in the small intestine but detected peptides at a low level in distal colon suggesting a low expression not detectable using immunohistochemistry or western blot (Figure 1E).

Due to discrepancies in the literature regarding the existence of a calcium-activated anion channel in the intestine that most likely corresponds to TMEM16A and the inherent limitation of the use of antibodies, we aimed to study this issue performing functional experimental approaches to try to understand the participation of TMEM16A on intestinal physiology. We included the breeding of an intestinal double knock-out mouse that lacks *Cftr* expression to test the role of TMEM16A on the intestine. This was based on the fact that intestinal *Cftr* silencing is sufficient to induce lethal obstructive episodes whose origin is due to attached mucus causing bacterial overgrowth (Hodges et al., 2008; Gustafsson et al., 2012; Schutte et al., 2014). We reasoned that in this altered intestinal environment the contribution of alternative anion channels are highly relevant in maintaining the secretory function after *Cftr* silencing. As expected, the intestinal silencing of *Cftr* drastically reduced anion secretion in ileum and proximal colon (Figure 3), that was accompanied with altered mucus properties and attachment (Figure 4). Attached mucus results in bacteria overgrowth and lethal intestinal infections in a significant fraction of animals (Figure 5) a phenomenon observed previously in this model (Hodges et al., 2008; Philp et al., 2018). On the contrary, intestinal silencing of *Tmem16a* had no effect on CCh or ATP induced calcium mediated anion secretion in ileum or proximal colon (Figure 3) and concomitantly did not affect mucus homeostasis or induce lethality in the animals, discarding a role for TMEM16A in epithelial anion secretion or bacterial overgrowth. As observed also in Figure 5, we induced the whole-body silencing of TMEM16A in the adult mouse using the tamoxifen inducible recombinase expression, and found that animals were lethally affected. This indicates that TMEM16A silencing induce a severe lethal phenotype which is different to the previously observed effects on airway development in the *Tmem16a*^-/-^ animal, and where intestinal TMEM16A unrelated (Rock et al., 2008).

It has been recently published that TMEM16A is essential for mucus secretion in the intestine and airways and that there is a higher level of expression of the protein in goblet cells (Benedetto et al., 2018). Our proteomic results show that the TMEM16A protein was found in low amounts in both goblet and non-goblet cells of the colon. We also observed that mucus architecture in both inner and outer mucus layers are clearly seen undisturbed in the *Tmem16a* deficient tissues but are severely altered in the *Cftr*^fl/fl^/*Villin*^cre/-^ mouse (Figure 4A,B), suggesting that TMEM16A is not required for normal mucus homeostasis in colon.

Finally, we asked if the remaining anion secretion observed in our intestinal *Cftr*-deficient animals was due to TMEM16A activity. As previously discussed this anion transport pathway is considered as a beneficial factor for the clinical course of the disease and related to a decreased frequency of intestinal obstructive episodes. As shown here in Figure 3, the silencing of *Tmem16a* in a *Cftr*^fl/fl^/*Villin*^cre/-^ background did not alter CCh or ATP-induced anion with respect to that observed in the *Cftr*^fl/fl^/*Villin*^cre/-^ mutant mouse, suggesting that TMEM16A does not participate in the remnant anion secretion seen in the absence of CFTR. It has been demonstrated that the remaining current in the colon of the *Cftr*^-/-^ mouse corresponds to bicarbonate secretion from goblet cells mediated by the activity of Bestrophin-2 (Yu et al., 2010). In agreement with Yu et al. (2010) observations, we detect a remaining current in the colon, but not in the ileum of the *Cftr*^fl/fl^/*Villin*^cre/-^ animals (Figure 3D). Although we have not tested cAMP involvement directly it is known that cAMP-induced anion secretion is not affected by *Tmem16a* silencing (Ousingsawat et al., 2009). More recently, Benedetto et al. (2017) observed that after intestinal silencing of *Tmem16a* there is a near total absence of cAMP and Ca^2+^ induced anion secretion. In addition, the same authors observe that intestinal *Tmem16a* inactivation led to an irregular and thinner mucus layer covering the colon epithelium (Benedetto et al., 2018). In contrast to this finding, we do not observe any changes in anion secretion and mucus layer organization in agreement with the absence of weight loss and lethality in these animals (Figure 5A). Surprisingly, there is no report of lethal intestinal obstruction in the mice used by Benedetto et al. (2017, 2018) as would have been expected from the severe secretory deficiency and mucus accumulation they report. Their contention that inhibition of TMEM16A might help to decrease mucus release and thus overcome obstruction is not upheld by the unaltered lethality we observe in our *Cftr*^fl/fl^/*Villin*^cre/-^ mice after additional *Tmem16a* silencing (Figure 5A). Future studies that allow the specific silencing of *Tmem16a* from goblet cells are required to elucidate the present differences.

But what is that *Tmem16a* silencing decreases weight gain in the *Cftr*^fl/fl^/*Villin*^cre/-^ mice? It might be possible that TMEM16A is expressed in an exclusive and minor subset of epithelial cells, explaining the TMEM16A positive signal found by the proteomics, responsible of controlling subtle nutrient uptake but unrelated to anion secretion. Since this “fail to thrive” phenotype of TMEM16A is only evident after *Cftr* silencing, it suggests that CFTR can replace TMEM16A in this function, but this is not occurring the other way round. Another possibility is that TMEM16A is expressed exclusively early after birth and surmounted later by CFTR in the intestine of the mouse, while in older animals housing conditions, microbiota or the genetic background of the animals could have an impact on the transport properties of the intestine and explain in part some of the differences observed in intestinal TMEM16A function across different publications (Flores et al., 2010).

Soon after its identification as the calcium-activated chloride channel, TMEM16A became a “hot topic” in epithelial physiology, particularly in airways and intestine. While its role in airway epithelium was fast elucidated and still remains unequivocally as a calcium-activated anion channel located in the apical membrane and allowing chloride and possibly bicarbonate secretion (Scudieri et al., 2012; Gorrieri et al., 2016), its role in the intestine has been hampered by contradictory results (Ousingsawat et al., 2009; Rock et al., 2009; He et al., 2011; Namkung et al., 2011a). While some of these inconsistencies can be explained by the use of inhibitors that are not so specific like niflumic acid that blocked calcium entry in the cells (Balderas et al., 2012) or the CaCC_inh_-A01 inhibitor, a molecule that also can block CFTR (Gianotti et al., 2016), results using animal models are more difficult to reconcile. While some evidence relates TMEM16A functioning as an apical anion channel secreting chloride to the gut lumen in the same fashion as it had been observed in the airways (Ousingsawat et al., 2009; Rottgen et al., 2018), some evidence points toward a basolateral localization where TMEM16A is regulating calcium-activated chloride secretion indirectly through the maintenance of calcium store release from the endoplasmic reticulum (He et al., 2011; Benedetto et al., 2017). Our own exploration of TMEM16A function on the intestine, carried out in different laboratories, showed that TMEM16A do not participate in calcium-dependent intestinal anion secretion or in intestinal mucus homeostasis. There is still need for further studies of the role of TMEM16A in intestinal epithelial function.

## Data Availability

The raw data supporting the conclusions of this manuscript will be made available by the authors, without undue reservation, to any qualified researcher.

## Ethics Statement

All experimental procedures were approved by the Centro de Estudios Científicos (CECs) Institutional Animal Care and Use Committee (Protocol N° 2015-01). The animal studies were carried out in accordance with the approved guidelines; and were reviewed and approved by the licensing and ethical committee of IRCCS San Martino–IST and by the Italian Ministry of Health. All animal experimental procedures were in full compliance according to Swedish animal welfare and legislation and approved by the Swedish Laboratory Animal Ethical Committee in Gothenburg, Sweden (number 73-15).

## Author Contributions

GV, AG, MJ, LA, BM-A, EN, PS, NP, PM-B, AP, and CF performed and designed the experiments. GV, AG, MJ, PS, NP, LG, GH, and CF analyzed the data and designed the figures. LG and CF conceived the research. CF wrote the manuscript. GV, MJ, PS, NP, LG, GH, and CF discussed the results, and corrected and approved the final version of the manuscript.

## Conflict of Interest Statement

The authors declare that the research was conducted in the absence of any commercial or financial relationships that could be construed as a potential conflict of interest.
