# Corrigendum: Normal Calcium-Activated Anion Secretion in a Mouse Selectively Lacking TMEM16A in Intestinal Epithelium

**DOI:** 10.3389/fphys.2019.01040

**Published:** 2019-08-27

**Authors:** Génesis Vega, Anita Guequén, Malin E. V. Johansson, Liisa Arike, Beatriz Martínez-Abad, Elisabeth E. L. Nyström, Paolo Scudieri, Nicoletta Pedemonte, Pamela Millar-Büchner, Amber R. Philp, Luis J. Galietta, Gunnar C. Hansson, Carlos A. Flores

**Affiliations:** ^1^Centro de Estudios Científicos (CECs), Valdivia, Chile; ^2^Universidad Austral de Chile, Valdivia, Chile; ^3^Department of Medical Biochemistry, University of Gothenburg, Gothenburg, Sweden; ^4^Telethon Institute of Genetics and Medicine (TIGEM), Pozzuoli, Italy; ^5^Istituto Giannina Gaslini, Genoa, Italy; ^6^Department of Translational Medical Sciences (DISMET), University of Naples Federico II, Naples, Italy

**Keywords:** TMEM16A, cystic fibrosis transmembrane conductance regulator, epithelial transport, colon, intestinal mucus

“Elisabeth E. L. Nyström” was not included as an author in the published article. The corrected ***Author Contributions*** statement appears below.

“GV, AG, MJ, LA, BM-A, EN, PS, NP, PM-B, AP, and CF performed and designed the experiments. GV, AG, MJ, PS, NP, LG, GH, and CF analyzed the data and designed the figures. LG and CF conceived the research. CF wrote the manuscript. GV, MJ, PS, NP, LG, GH, and CF discussed the results, and corrected and approved the final version of the manuscript.”

The authors apologize for this error and state that this does not change the scientific conclusions of the article in any way. The original article has been updated.

