# Supplementary material for: Normal Calcium-Activated Anion Secretion in a Mouse Selectively Lacking TMEM16A in Intestinal Epithelium
Source: Front Physiol. 2019 Jun 13;10:694. doi: 10.3389/fphys.2019.00694 (PMC6585864; doi:10.3389/fphys.2019.00694)
Supplement: Supplementary file 1 [file Data_Sheet_1.pdf]

Supplemental Figure 1.

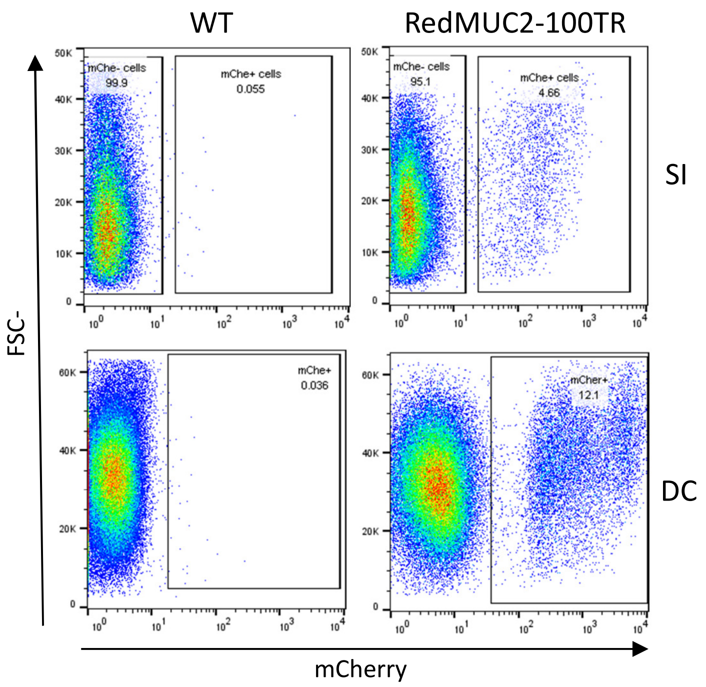

Supplemental Figure 1. Isolated epithelial cells were sorted by FACS from the live cell population, determined by Fixable Viability Dye eFluor™ 780 staining, on expression of mCherry-MUC2. The positive gate was set in the 610/20 (561) channel with WT epithelial cells not expressing mCherry-MUC2 as control. The proportion of goblet cells were 4.7% in the small intestine and 12% in colon.

Supplemental Figure 2.

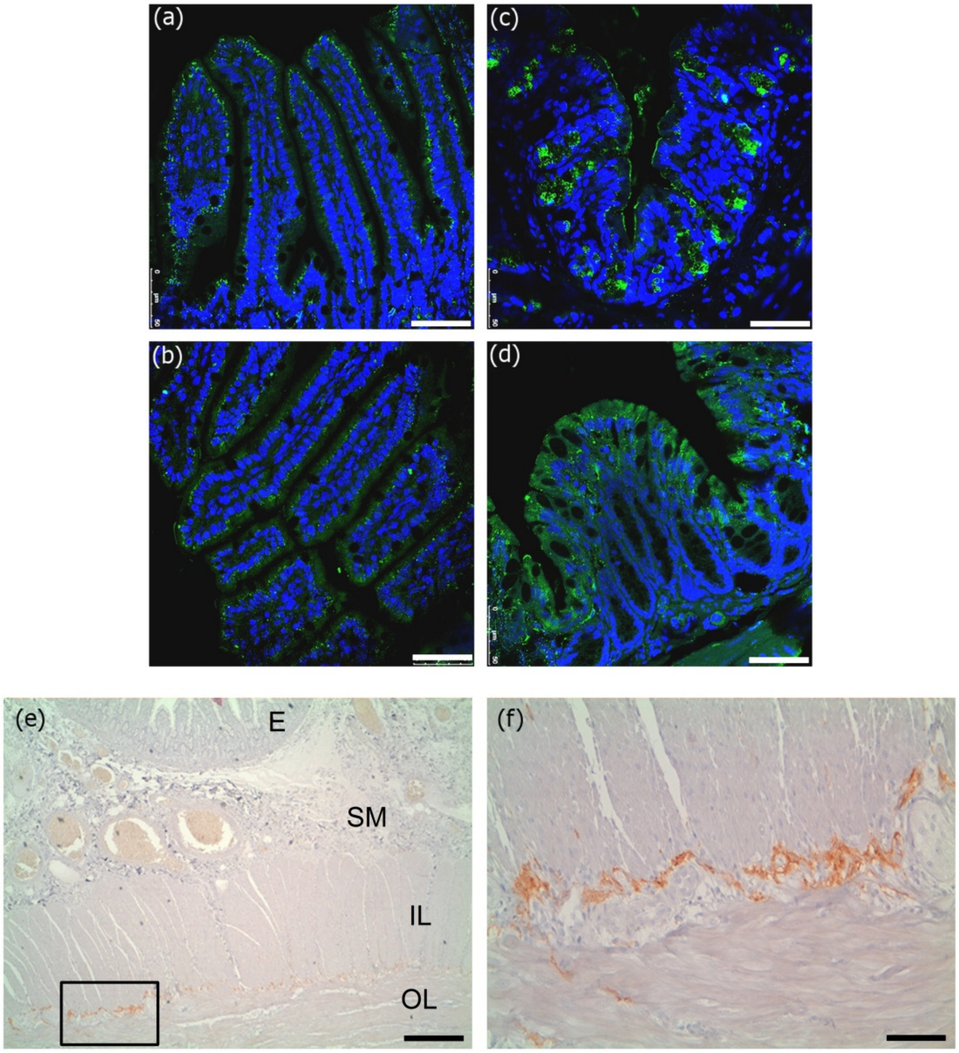

Supplemental Figure 2. Intestinal immunilocalization of TMEM16A. (a) and (b) Ileum samples show intracellular staining mostly with few cells having apical membrane staining unrestrictive of the inactivation of *Tmem16a*. Proximal colon samples of (c) wild type mice with apical membrane staining of surface cells and broader intracellular staining. (d) Samples still presented some cells with apical membrane and increased intracellular staining. (e) Human intestine presented intense reaction in cells corresponding to the enteric nervous system; E indicates epithelium, SM submucosa, IL inner circular muscular layer and OL outer longitudinal muscular layer. (f) Magnification of the area indicated in (e) showing TMEM16A positive cells. No epithelial cells were stained. Scale bars 50 µm (a-d), 100 µm (e) and 20 µm (f).

Supplemental Figure 3.

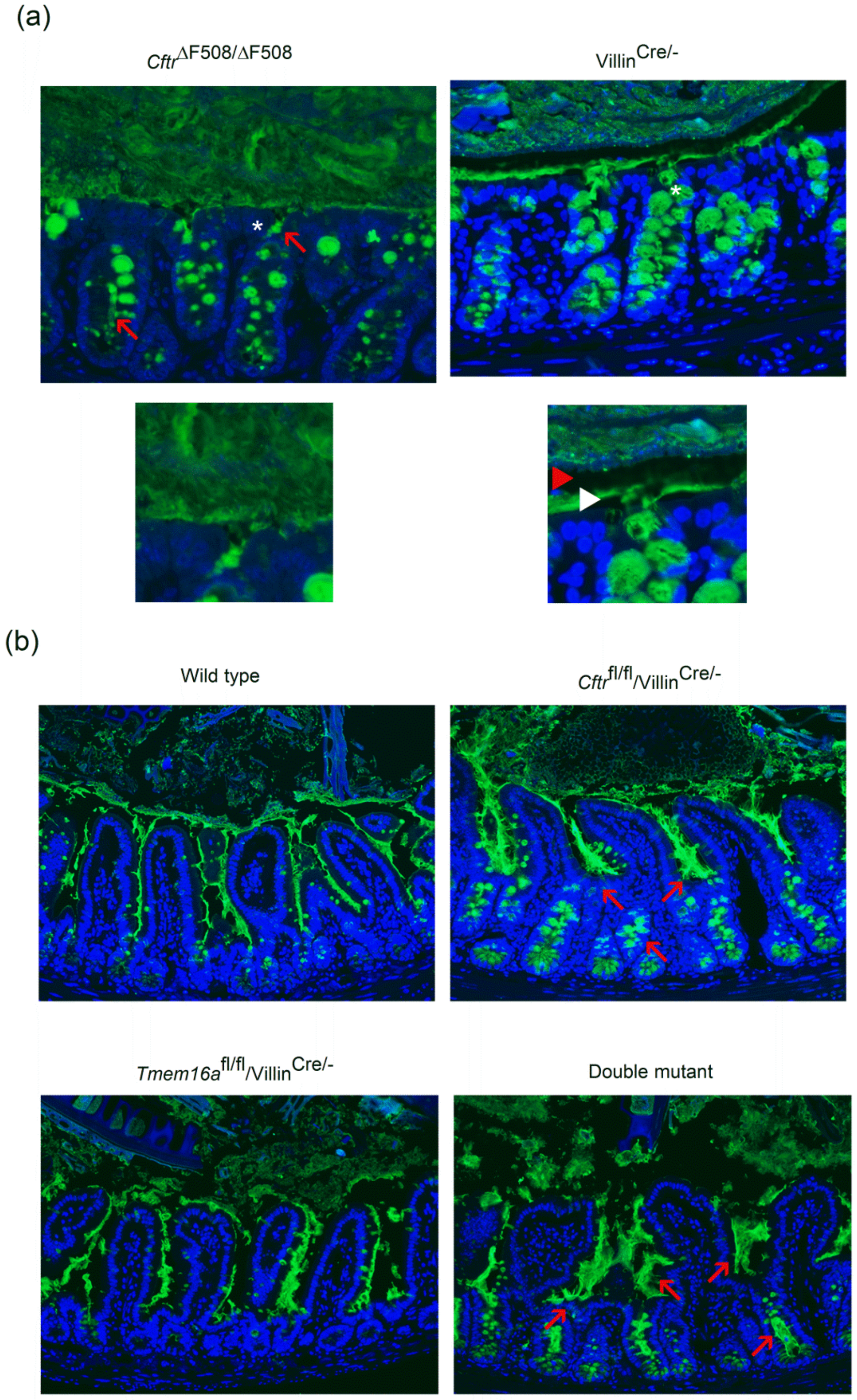

Supplemental Figure 3. Mucus structure is altered in colon of *Cftr*-mutant mouse but not in the *Villin*-cre mouse or after *Tmem16a* silencing in the Ileum. Representative images of MUC2

staining in Carnoy's fixed intestinal samples are shown in green. (a) corresponds to colon samples from *Cftr*$^{ΔF508/ΔF508}$ and *Villin*$^{Cre/-}$ Red arrows indicate massive release of mucus from the crypts and white asterisk indicate crypts amplified in the corresponding lower images. Lower images show detailed mucus structured in clearly defined external layer (red arrow head) and internal layer (white arrow head) as observed in the *Villin*$^{Cre/-}$ but the structure is lost in the*Cftr*$^{ΔF508/ΔF508}$ animals. (b) Ileum tissue samples. Red arrows indicate abnormal mucus. Blue staining correspond to Hoescht nuclear DNA; (n=5 for each group).

Supplemental Figure 4.

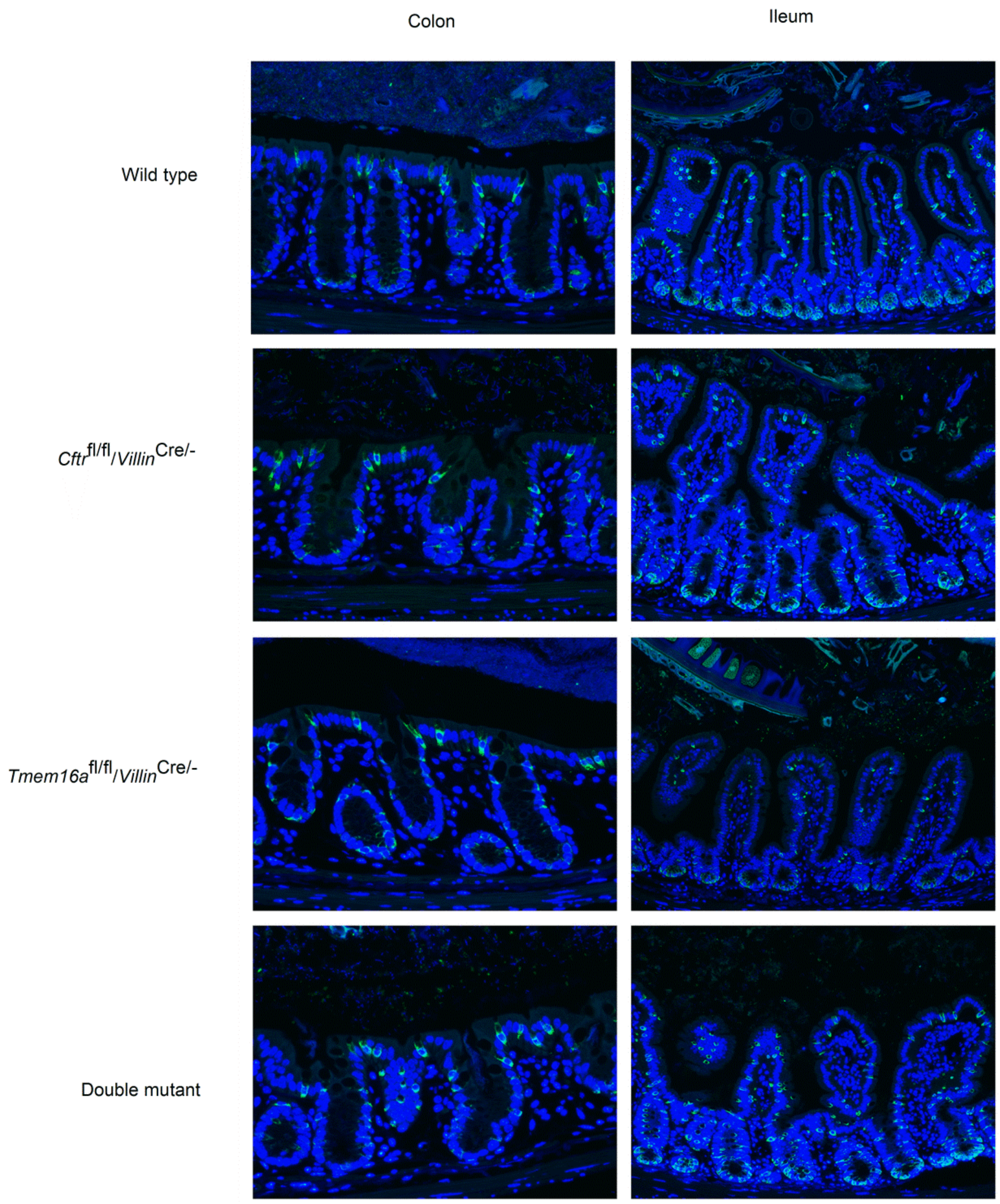

Supplemental Figure 4. Muc2-apomucin staining in colon and ileum of the mouse. Representative images of Muc2-apomucin staining in Carnoy's fixed intestinal samples are shown in green. Positive can be observed from bottom to the top of the crypts of the colon. Ileum presented positive cells on the villi. No obvious differences were detectable among genotypes; n=5 for each group.
